# Supplementary figures and images for: Generalizing to generalize: Humans flexibly switch between compositional and conjunctive structures during reinforcement learning
Source: PLoS Comput Biol. 2020 Apr 13;16(4):e1007720. doi: 10.1371/journal.pcbi.1007720 (PMC7179934; doi:10.1371/journal.pcbi.1007720)

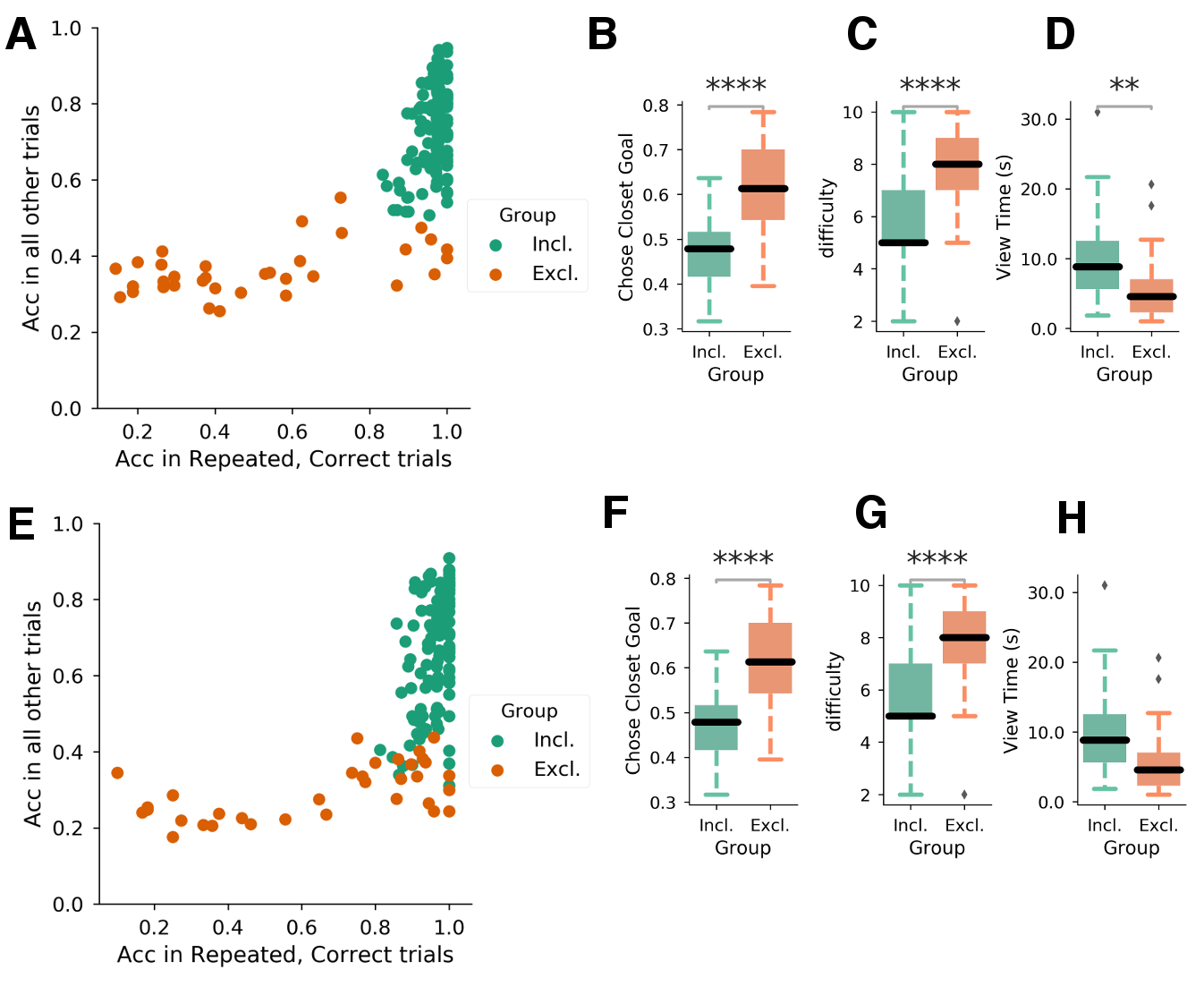

Supplement: S1 Fig — A,E: Accuracy in repeated, correct trails vs. all other trials. B,F: Proportion of time the closest goal was selected by inclusion status. C,G: Subject rated difficulty by inclusion status. D,H: Time spend reading viewing instructions by inclusion status. (TIF) [file pcbi.1007720.s001.tif]

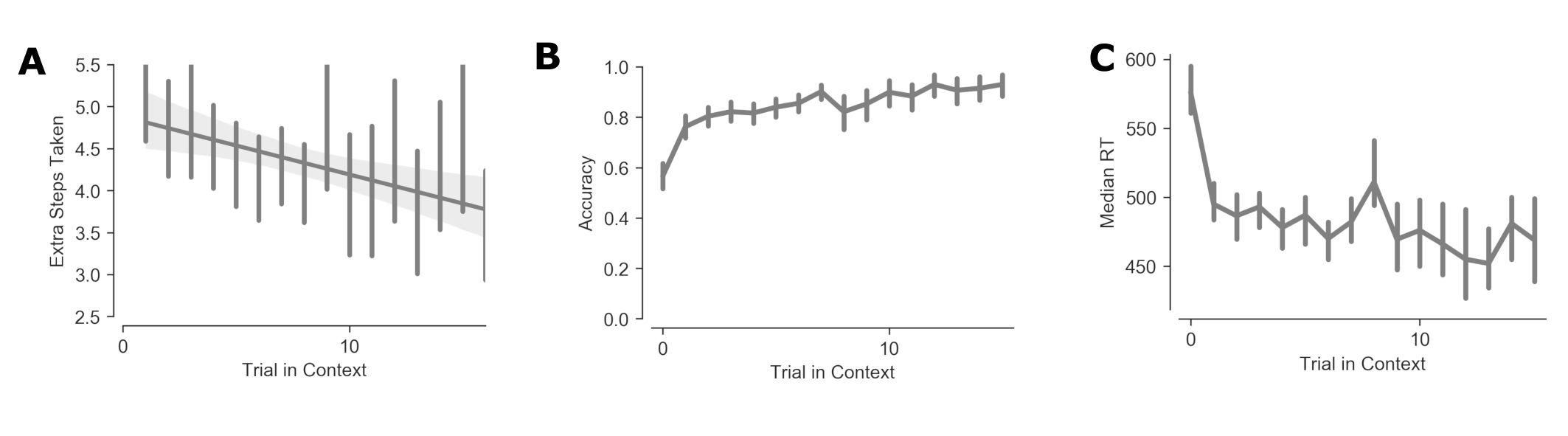

Supplement: S2 Fig — Accuracy (A), median reaction time (B) and the excess number of steps taken over the shortest path (C) shown as a function of the number of trials within each training context. (TIF) [file pcbi.1007720.s002.tif]

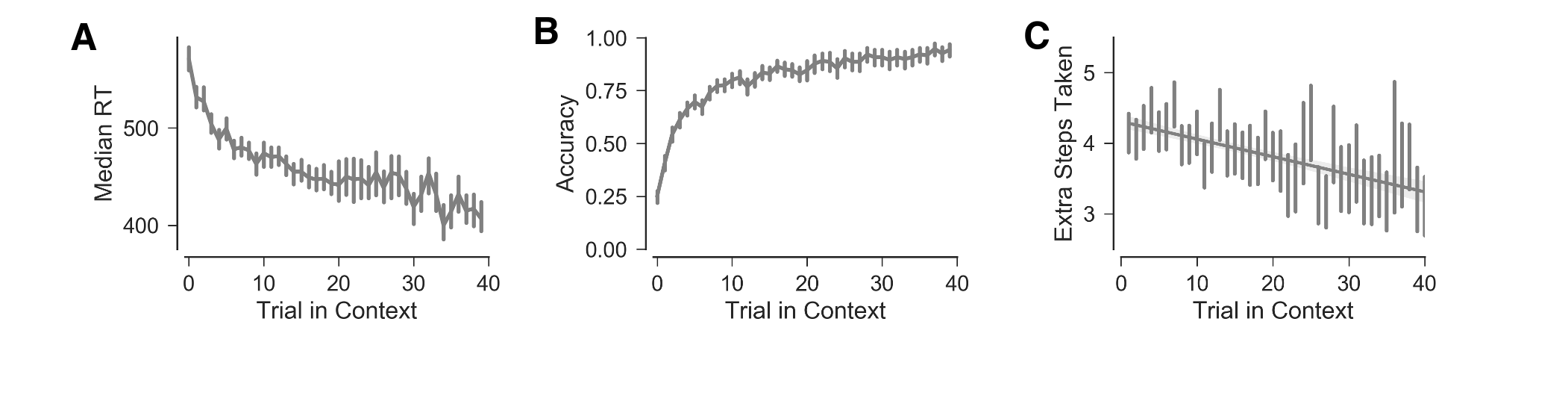

Supplement: S3 Fig — Accuracy (A), median reaction time (B) and the excess number of steps taken over the shortest path (C) shown as a function of the number of trials within each training context. (TIF) [file pcbi.1007720.s003.tif]

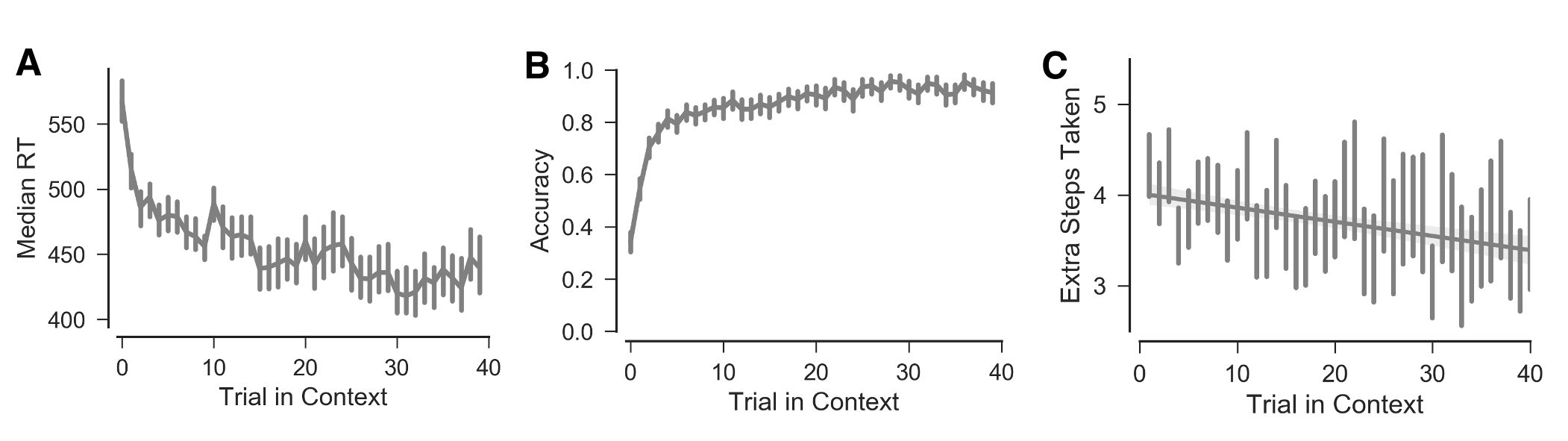

Supplement: S4 Fig — Accuracy (A), median reaction time (B) and the excess number of steps taken over the shortest path (C) shown as a function of the number of trials within each training context. (TIF) [file pcbi.1007720.s004.tif]

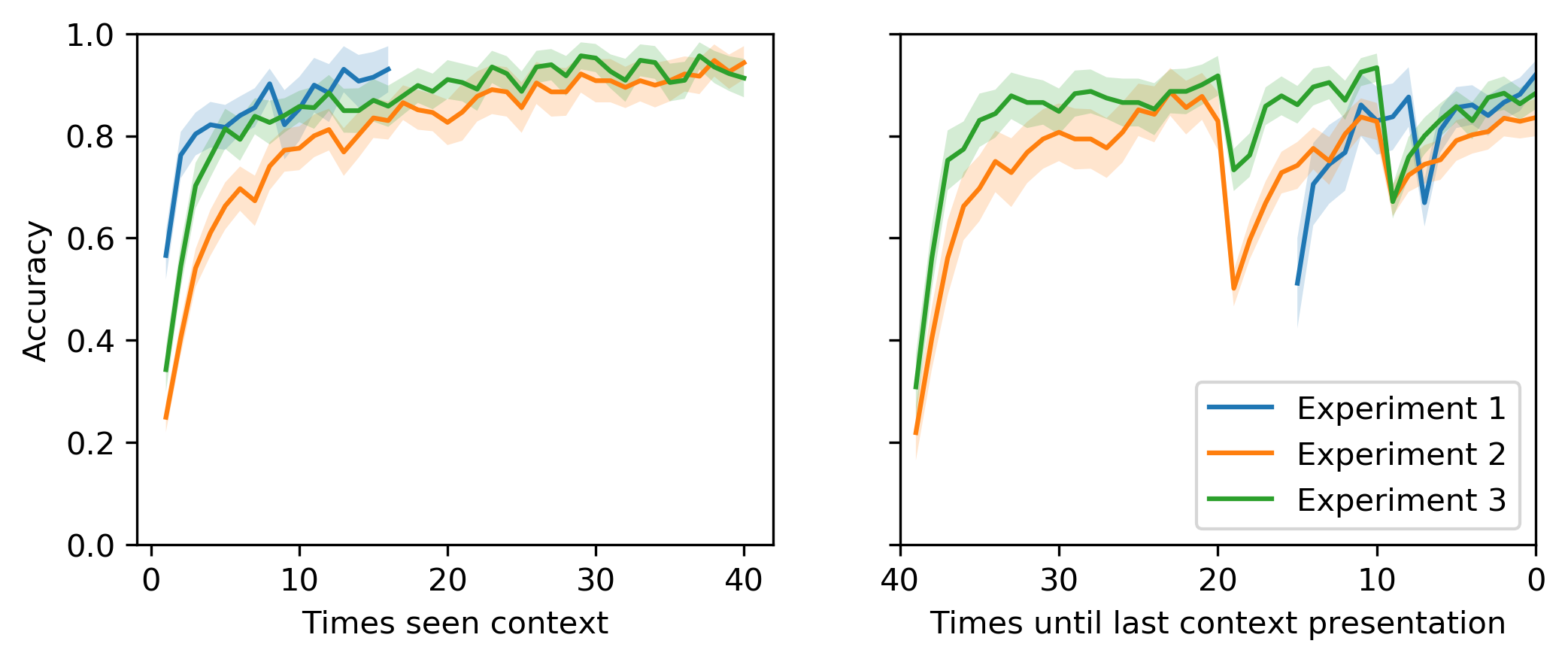

Supplement: S5 Fig — Left: Accuracy as a function of the number of presentations in each context. Initial differences reflect a difference in chance accuracy between experiments Right: Accuracy as a function of number of presentations remaining (per context) within the training phase. Sharp drops in accuracy reflect the fact that each context was not shown the same number of times. (TIF) [file pcbi.1007720.s005.tif]
