# Supplementary material for: Generalizing to generalize: Humans flexibly switch between compositional and conjunctive structures during reinforcement learning
Source: PLoS Comput Biol. 2020 Apr 13;16(4):e1007720. doi: 10.1371/journal.pcbi.1007720 (PMC7179934; doi:10.1371/journal.pcbi.1007720)
Supplement: S1 Table — The number of trials within each context is balanced such that each goal and each mapping is presented the same number of trials across both training and test. Subjects saw either the “Repeat Test” context or the “Switch Test” contexts. (PDF) [file pcbi.1007720.s006.pdf]

Context	Goal	Mapping	n Trials
Train 1	A	High Pop	8
Train 2	A	High Pop	8
Train 3	B	Low Pop	16
Repeat Test 1	A	High Pop	4
Repeat Test 2	A	High Pop	4
Repeat Test 3	B	Low Pop	8
Switch Test 1	A	Low Pop	4
Switch Test 2	A	Low Pop	4
Switch Test 3	B	High Pop	8
