# Supplementary material for: Generalizing to generalize: Humans flexibly switch between compositional and conjunctive structures during reinforcement learning
Source: PLoS Comput Biol. 2020 Apr 13;16(4):e1007720. doi: 10.1371/journal.pcbi.1007720 (PMC7179934; doi:10.1371/journal.pcbi.1007720)
Supplement: S3 Table — The number of trials within each context is balanced such that each goal and each mapping is presented the same number of trials across both training and test. (PDF) [file pcbi.1007720.s008.pdf]

Context	Goal	Mapping Popularity	n Trials
Train 1	A	High	10
Train 2	A	High	10
Train 3	A	Low	20
Train 4	B	High	40
Train 5	C	Low	40
Test 1	A	Low	10
Test 2	B	High	10
Test 3	C	High	5
Test 4	C	Low	5
