# Supplementary material for: Generalizing to generalize: Humans flexibly switch between compositional and conjunctive structures during reinforcement learning
Source: PLoS Comput Biol. 2020 Apr 13;16(4):e1007720. doi: 10.1371/journal.pcbi.1007720 (PMC7179934; doi:10.1371/journal.pcbi.1007720)
Supplement: S1 Text — (PDF) [file pcbi.1007720.s009.pdf]

S1: Subject Exclusion Criteria

Our subject pool was recruited online using Amazon's Mechanical Turk platform. In order to control for the possibility some subjects were not engaged in the task and instead rushed through it as quickly as possible, we used subject performance on the training contexts to identify non-performance on the task. In experiments 2 and 3, we used two measures of performance during training: (1) accuracy on trials in which the preceding trial shared the same context and in which the subject responded correctly and (2) the probability subject goal-selection accuracy was at chance in all other trials, according to a binomial model. A Gaussian mixture model were fit to these two measures, we allowed the number of clusters to range between one and three and allowed for the constraints over the covariance matrix to vary from spherical, diagonal, tied or full. Bayesian information criterion (BIC) was used for model selection, and the model with the lowest BIC was chosen. For both experiments 2 and 3, a model with 2 components and a diagonal constraint on the covariance matrix had the lowest BIC (Figure S1)

The members of the largest cluster were carried forward for analysis and all other subjects were used excluded. Follow up analyses found the excluded subjects were more likely to choose the goal closest to the initial location in each trial in both experiment 2 (Fig S1F; $t(65.8) = -6.4$, $p < 3 \times 10^{-8}$) and experiment 3 (Fig S1B; $t(42.8) = -7.1$, $p < 9 \times 10^{-9}$). In experiment 3, excluded subjects tended to rate the experiment as more difficult (Fig S1C; $t(47.6) = -4.3$, $p = 9 \times 10^{-5}$) and spent significantly more time viewing the instructions (Fig S1D; $t(45.1) = 3.5$, $p = 0.0011$). Neither of these two effects were present in experiment 1 (Fig S1G, H; difficulty: $t(53.3) = 0.76$, $p > 0.4$, viewing time: $t(57.7) = 0.30$, $p > 0.7$)

A similar clustering procedure was used to determine the subjects in experiment 1 that were carried through for further analysis but with loosened criteria as a large number of subjects were found to be excluded using the criteria above. This was likely due task differences as experiment three was substantially easier that the other two tasks. Accuracy in trials in which the context was sequentially repeated was again used as a dimension of the clustering analysis. In lieu of the binomial probability that all other trials were at chance accuracy, the raw accuracy in these trials was used. Chance accuracy in experiment 3 was 50%, and this was found to be too strict of a criterion. Subjects excluded in were less likely to choose the closest goal closest to the initial location of the agent ($t(115, 5) = -1.99, p = 0.049$) but we saw no difference in the time spent viewing instructions ($t(150.6) = 0.82, p > 0.4$, cohen's d $= 0.87$) or in rated task difficulty ($t(106.5) = -0.29, p > 0.7$)
