# Supplementary material for: Generalizing to generalize: Humans flexibly switch between compositional and conjunctive structures during reinforcement learning
Source: PLoS Comput Biol. 2020 Apr 13;16(4):e1007720. doi: 10.1371/journal.pcbi.1007720 (PMC7179934; doi:10.1371/journal.pcbi.1007720)
Supplement: S2 Text — (PDF) [file pcbi.1007720.s010.pdf]

S2: Statistical analysis of training contexts

While performance in the training context was not directly of interest, generalization depends on learning the statistics of the training environment. We assessed three measures of learning as a function of time in the training contexts: goal-choice accuracy, reciprocal reaction-time and navigation efficiency, where navigation efficiency was defined as the number of steps taken in a trial minus the minimum path length required to reach the chosen goal. Each measure was analyzed using a hierarchical Bayesian general linear model:

$$f(x) = \alpha_{\text{subj}} + \beta_t * t + \beta_t t + \beta_{\text{rep}}\text{rep} \tag{16}$$

where x is the measure of interest, f is the linking function, t is the number of trials observed within a context, rep $\in 0, 1$ is an indicator function for whether the previous trial was correct and shared the same context and α_{subj} is a subject specific bias term (Kruschke, 2014). For accuracy, we used a logistic function as a linking function, for reciprocal reaction time we use a linear linking function (Noorani & Carpenter, 2016), and for navigation efficiency we use a Poisson linking function.

For the accuracy and reaction time analysis, we further assumed the hierarchical priors $\alpha_{\text{subj}} \sim \mathcal{N}(\mu_\alpha, \sigma_\alpha)$, $\beta_t \sim \mathcal{N}(0, 100)$ and $\beta_{\text{rep}} \sim \mathcal{N}(0, 100)$, with the weakly informative hyper-prior $\mu_\alpha \sim \mathcal{N}(0, 100)$, $\sigma_\alpha \sim \mathcal{N}(0, 100)$. For the navigation efficiency analysis, we used the stronger priors of $\beta_t \sim \mathcal{N}(0, 10)$ and $\beta_{\text{rep}} \sim \mathcal{N}(0, 10)$ as priors, with the weakly informative hyper-prior $\mu_\alpha \sim \mathcal{N}(0, 5)$, $\sigma_\alpha \sim \mathcal{N}(0, 5)$ as the less informative priors failed to converge, likely due to the Poisson link function.

Non-zero values of β_t were interpreted as learning across time while all other predictors were considered nuisance parameters. The analysis of goal accuracy was also used to compare experiments, with the hierarchical mean over subject intercepts, μ_α, as a metric of group-level accuracy in the task.
