# Supplementary material for: Generalizing to generalize: Humans flexibly switch between compositional and conjunctive structures during reinforcement learning
Source: PLoS Comput Biol. 2020 Apr 13;16(4):e1007720. doi: 10.1371/journal.pcbi.1007720 (PMC7179934; doi:10.1371/journal.pcbi.1007720)
Supplement: S3 Text — (PDF) [file pcbi.1007720.s011.pdf]

S3: Comparison between experiments

In this section, we examine the degree to which goals and mappings are related in the training contexts. We formalize this with normalized mutual information (NMI), which we define as

$$\text{NMI} = 1 - \frac{H(G|M)}{H(G)} \tag{17}$$

where $H(G|M)$ is the conditional entropy of the goals given the mapping and $H(G)$ is the marginal entropy of the goals. Both the conditional and marginal entropy are defined in bits as

$$H(G) = -\sum_{g} p_g \log_2 p_g \tag{18}$$

and

$$H(G|M) = -\frac{1}{N_m} \sum_{g,m} p_{g|m} \log_2 p_{g|m} \tag{19}$$

where p_g is defined as the frequency goal g is the correct goal in the training context, $p_{g|m}$ is the frequency goal g is the correct goal conditioned on mapping m and N_m is the total number of mappings.

For example, in experiment 1, there are three training contexts, two of which have "A" as the correct goal and one of which has "B." Therefore, $H(G) = -\left(\frac{2}{3}\log_2\frac{2}{3} + \frac{1}{3}\log_2\frac{1}{3}\right) \approx$ 0.92bits. Because there is a perfect correspondence between mappings and goals in experiment 1, $H(G|M) = 0$ and consequently, NMI=1.
