# Supplementary material for: Generalizing to generalize: Humans flexibly switch between compositional and conjunctive structures during reinforcement learning
Source: PLoS Comput Biol. 2020 Apr 13;16(4):e1007720. doi: 10.1371/journal.pcbi.1007720 (PMC7179934; doi:10.1371/journal.pcbi.1007720)
Supplement: S4 Text — (PDF) [file pcbi.1007720.s012.pdf]

S4: Non-contextual agent

As a final control model, we also simulated a non-contextual Q-learning agent that aggregated the reward values over all trials. This was done to control for potential frequency effects. Previous work on context-based generalization in a bandit setting has provided evidence for context-popularity based generalization over frequency-based generalization, including the prior in the very first trials(Collins & Frank, 2016), and building on this work, we do not expect to find frequency effects. Moreover, the task was designed to balance goal reward frequency, such that each goal is rewarded the same number of times across in each experiment. Nonetheless, subjects (and the models) may not experience the veridical task statistics due to variations in their choice behavior. Thus, we also evaluated a Q-learning agent that aggregated reward values across all of the trials in each experiment, learning a single set of Q-values for all of the contexts. This Q-learning agent is otherwise identical to our previously described Q-learner and was simulated as previously described.

Unsurprisingly, this context-free Q-learning agent did not perform above chance in any of the three tasks (Training Accuracy, Exp 1: 0.42 Exp2: 0.24, Exp3: 0.33; Test Accuracy Exp 1: 0.44, Exp 2: 0.23 Exp 3: 0.30). Nor was the model able to account for any of the principal findings described above. In experiment 1, There was no significant effect of task (HPD = [-0.118, 0.111]) or goal (HPD =[-0.110, 0.219]). In experiment 2, there was no significant effect of $1 > 2$ (HPD = [-0.095, 0.07]) or $3 > 4$ (HPD = [-0.076, 0.203]. In experiment 3, there was no significant effect of $1 > 4$ (HPD = [-0.092, 0.121]) or $2 > 3$ (HPD = [-0.091, 0.121]).
