# Supplementary material for: Generalizing to generalize: Humans flexibly switch between compositional and conjunctive structures during reinforcement learning
Source: PLoS Comput Biol. 2020 Apr 13;16(4):e1007720. doi: 10.1371/journal.pcbi.1007720 (PMC7179934; doi:10.1371/journal.pcbi.1007720)
Supplement: S5 Text — (PDF) [file pcbi.1007720.s013.pdf]

S5: Meta-Generalization vs. Mixture of subjects

The results presented for experiment 3 could have plausibly been either individual subjects adopting a meta-generalization strategy or a mixture of subjects performing only one strategy. In order to assess this, we examined the difference scores for individual subjects to see if there was a relationship between a subjects tendency to show the independent effect (greater accuracy in test context 1 than 4) and their tendency to show the joint effect (greater accuracy in test context 2 than 3).

We also examined the relationship between the two difference scores in the model, comparing the meta-learning agent to an equal mixture of joint and independent clustering agents. As in all of our simulations above, we drew 200 batches of simulated tasks samples from a pool of 2500 and match the sample size to that of human subjects.

We first looked for a correlation between the two difference scores. Intuitively, we would expect a negative relationship between the difference scores in a mixture of agents, as an agent would either show one effect or the other. This bears out in our modeling where there was a negative correlation in the mixtures of joint and independent agents (mean $r = -0.17$), with 98.5% of batches showing this negative correlation. Surprisingly, there was also a modest negative correlation in our batch of the meta-agent (mean $r = -0.07$), but this effect was not statistically reliable as 77.5% of batches had r-values below zero. Our human subject data, in contrast, showed a mild positive correlation that was not statistically distinguishable from zero ($r = 0.04$, $p > 0.3$), thus presenting a null result for the mixture of joint and independent agents.
