# Supplementary material for: Generalizing to generalize: Humans flexibly switch between compositional and conjunctive structures during reinforcement learning
Source: PLoS Comput Biol. 2020 Apr 13;16(4):e1007720. doi: 10.1371/journal.pcbi.1007720 (PMC7179934; doi:10.1371/journal.pcbi.1007720)
Supplement: Attachment — Submitted filename: Response To Reviewers.pdf [file pcbi.1007720.s015.pdf]

Dear Dr. O'Reilly and Dr. Blackwell,

Thank you for your consideration of our work. My colleague and I have prepared a revised manuscript and hereby resubmit our manuscript "Generalizing to generalize: humans flexibly switch between compositional and conjunctive structures during reinforcement learning" for consideration for publication.

We would also like to thank the reviewers for their encouraging and helpful comments. We have made the following changes to the manuscript to address their concerns:

1. We have added analysis of a non-contextual bandit to the supporting information, please see section "Non-contextual agent"
2. We have added additional simulations and behavioral analysis to identify whether different pools of subjects adopted different strategies in experiment 3. These are included in a new section in the supporting information, "Meta-Generalization vs. Mixture of subjects"

In addition, we have revised the manuscript to address individual points raised by reviewers. We include a detailed response to these below.

Sincerely,

Nicholas T Franklin   Michael J Frank

Reviewer #1:

This paper investigates whether humans adaptively change their generalization strategies depending on environmental statistics. More specifically, the hypothesis is that participants will change the degree to which they blend "joint" and "independent" clustering of the task-relevant statistics (action set and reward function). The paper describes a series of three experiments which vary in the degree to which participants should favour the joint (exp 1), independent (exp 2) or meta-generalization (exp 3) strategies. The results show that humans are biased to generalize even when this is not advantageous, with the models further indicating a meta-generalization strategy.

The study is designed to address an important and timely question: What are the mechanisms which enable humans to flexibly generalize behaviour to novel situations? The tasks are well-designed, and the authors take great care to rule out lower-level confounds. The simulations are done very carefully with state of the art techniques. Overall, I think this is a well-crafted manuscript with nothing major standing in the way of publication.

That being said, however, I think the paper in its current form under-utilizes the rich behavioural data from the training and test conditions. I have a few suggestions which I think might ultimately aide understanding of the paper's conclusions and methods and provide richer information about human behaviour than from the mostly qualitative model comparison.

We thank the reviewer for their helpful feedback and encouraging comments.

1. A key component of the model in the paper is the clustering which is done via a Chinese Restaurant Process. This made me wonder how essential this component is to explain the data. It makes the key prediction that accuracy and learning speed are influenced by context popularity, rather than frequency of trials. However, I don't see the alternative "frequency-based" models tested – the flat models do not work across contexts, so ask a different question. I wondered to what extent a Q-learning agent which averages over all trials would predict participant's behaviour. For instance, the Q-learner might average across all of participants' trajectories (rather than contexts) and thus make different predictions to a clustering model based on "popularity". Even though each goal was rewarded the same amount of trials, participants' choices likely did not reflect this (~75-80% performance). In this sense, a goal that was more frequently visited (even when unrewarded) might be chosen with higher likelihood in the test conditions. I don't expect this model to do very well, but I nevertheless think it would be an appropriate control model that would add additional confidence in the results.

We agree with the reviewer that this is a useful control. We have added the following text as an appendix ("Non-contextual agent"):

On page 8:

Note also that all of the generalizing and non-generalizing models learn contextually: the task was designed to require context and aggregating reward across all trials regardless of context leads to chance performance and does not produce any of the qualitative behaviors discussed below (see SI: Non-contextual agent*).*

SI: Non-contextual agent:

As a final control model, we also simulated a non-contextual Q-learning agent that aggregated the reward values over all trials. This was done to control for potential frequency effects. Previous work on context-based generalization in a bandit setting has provided evidence for context-popularity based generalization over frequency-based generalization, including the prior in the very first trials (Collins and Frank, 2016), and building on this work, we do not expect to find frequency effects. Moreover, the task was designed to balance goal reward frequency, such that each goal is rewarded the same number of times across in each experiment. Nonetheless, subjects (and the models) may not experience the veridical task statistics due to variations in their choice behavior. Thus, we also evaluated a Q-learning agent that aggregated reward values across all of the trials in each experiment, learning a single set of Q-values for all of the contexts. This Q-learning agent is otherwise identical to our previously described Q-learner and was simulated as previously described.

Unsurprisingly, this context-free Q-learning agent did not perform above chance in any of the three tasks (Training Accuracy, Exp 1: 0.42 Exp2: 0.24, Exp3:

0.33; Test Accuracy Exp 1: 0.44, Exp 2: 0.23 Exp 3: 0.30). Nor was the model able to account for any of the principal findings described above. In experiment 1, There was no significant effect of task (HPD = [-0.118, 0.111]) or goal (HPD =[-0.110, 0.219]). In experiment 2, there was no significant effect of 1 > 2 (HPD = [-0.095, 0.07]) or 3 > 4 (HPD = [-0.076, 0.203]. In experiment 3, there was no significant effect of 1 > 4 (HPD = [-0.092, 0.121]) or 2 > 3 (HPD = [-0.091, 0.121]).

2. I wondered in how far the first few button presses (rather than a trial overall) could be indicative of generalization strategy. First, did participants show a preference for either one of the hands on the first button press? I ask this, because some mappings were more popular on a context level (though not on overall trials), so we might expect participants to be biased in some way. More generally, participants might have acted consistent with a) navigating to the most popular goal and b) the mapping for which this goal was more popular, e.g. in experiment 2, we might expect them to use the left hand, because goal A was more frequent overall and more frequently associated with the left hand. Thus, a joint-clustering agent should be biased to "try" the mapping with higher joint likelihood. After this initial button press, participants can reason conditional on the correct mapping. Thus, if the chosen mapping was correct, participants should continue navigating towards the same goal. However, if the mapping was incorrect, it would then be the second button press which would distinguish joint from independent clustering. If participants then press the button (on the other hand) which corresponds to the same direction as their first button press, then they likely clustered independently (still navigating to the same goal), but if they choose a different direction they likely clustered jointly (on cases where the two mappings have different goal popularities, e.g. exp 1 and 3). Perhaps there is not a lot of data to work with for this, but I think it would give a strong indication of the strategies employed by participants.

We thank the reviewer for the suggestion and we agree the suggested analysis is an additional prediction that differentiates joint and independent clustering.

Unfortunately, we are probably underpowered in detecting this effect in our data due to the small number of trials. Using Bayesian logistic regression, we compared experiment 1 (Joint) and experiment 2 (Independent) on the number of times subjects pressed a key for the same direction given that they switch mappings in sequential trials. We find that subjects do indeed choose the same directions more often in experiment 2 than experiment 1, as the reviewer suggested, but this effect was not strong statistically (one-tailed p-value = 0.03, HPD = [-0.015, 0.24]). This analysis is further complicated by the fact that subjects see far more trials in experiment 2 than experiment 1, meaning that any effect we do see may be driven by a better understanding of the mappings.

We have included the following paragraph in the Limitations on page 24:

Relatedly, we largely focus on the consequences of joint and independent clustering on goal generalization in our experimental design, and have not considered how joint and independent clustering make different predictions about how mappings are generalized. For example, if an independent clustering

agent tries a response from the wrong mapping on the first trial of a new context, we would expect the agent to try to move in the same direction on the next trial using the corresponding response of a different mapping. We would not expect this tendency from a joint agent, because it would presumably use the observed mapping information to adjust its high-level plan. While our experiment was not designed to find this effect within-subjects, we can nonetheless look for this pattern of behavior in a cross experiment comparison. Using Bayesian logistic regression, we compared the tendency of subjects to ``switch'' from one mapping to another following an unsuccessful attempt to move, that is, move in the same direction as they would have in a previous trial had the previous mapping been valid for the context. We would expect this to be more common in experiment 2, as the task supports independent clustering, than in experiment 1, where the task supports joint clustering. While we find this prediction is directionally correct, we do not find strong statistical evidence for it (E[\beta_{exp2>exp}]=0.128, one-tailed p-value = 0.03, HPD = [-0.015, 0.24]), in a Bayesian logistic regression that considers both training and test contexts and includes trial number, the number of trials within a context, whether a context was repeated sequentially, and an individual subject bias term as nuisance regressors. One potential interpretation is that this follow-up analysis across experiments is simply underpowered. Further complicating this analysis is a difference in experience with the task in experiment 1 and experiment 2, which we attempt to control for statistically. As such, we can not draw strong conclusions from our data about how mapping generalization differentiates joint and independent clustering.

3. A similar point relates to how well each strategy predicts behaviour in the training condition. One of the paper's central claims is that humans adaptively adjust their generalization strategies according to the task statistics. In this sense, the models should make predictions about behaviour in, say, context 3, after having seen contexts 1 and 2 for some limited amount of time (due to the autocorrelation). If the strategies are adaptive, one might expect participants behaviour to be gradually better predicted (even within training) by one strategy than the others.

This is true in principle, but in earlier pilot experiments, we found it difficult to detect generalization effects during the early training phase. In general, the effect sizes are a lot smaller than the train/test design in the manuscript, presumably because there is far less evidence for one set of task statistics over another without sufficient experience. This is consistent with previous findings, where people don't tend to show evidence for generalization within training when contexts can be clustered as it takes time to learn the overall structure (Collins and Frank, Cognition, 2016). More germane, in our design, we sacrifice any ability to resolve generalization during training and focused on identifiability in the test phase.

We have made the following note in the manuscript on page 23:

The experiments presented here were designed to show the effects of joint and independent clustering on goal generalization during a defined generalization

phase and did not examine other aspects of generalization, including generalization during training, interactions with planning, and mapping generalization. Detecting generalization during training can be difficult, as subjects have to simultaneously learn task structure and associative contingencies. Previous related work has found clear evidence for generalization effects only after sufficient training experience was accrued to infer the most likely structure that affords context clustering (Collins and Frank, 2016). In the experiments presented above, joint and independent clustering are not distinguishable during training in the sample sizes we have collected.

4. Did the models also take into account progression through the test conditions? This would change goal popularity and would lead to different predictions about behaviour – which might partly explain the different pattern of participants' goal choice in experiment 2 (Fig 7c and d).

Yes. We have clarified this point on page 8:

The key difference between the generalizing and non-generalizing agents is that the generalizing agents pool information across contexts, whereas the non-generalizing agents do not. This process continues throughout all trials, such that the new contexts encountered by the generalizing agents during the test phase can influence subsequent generalization.

Minor:

1. How likely were participants to simply repeat their previous choice of goal in a new context? Does including this regressor at all affect the model parameters in the logistic regression and the Bernoulli analysis? It seems quite likely that in new contexts there would be at least some "stickiness" to participants' choices which gives way via reinforcement to the correct goal over time, but perhaps dominates somewhat on the first trial in any new test condition.

We have changed the nuisance regressors in our statistical analysis from including an indicator for whether a context is repeated to whether the interaction of whether a context is repeated and the previous goal choice is correct. This adds "stickiness" to the participants' choices and does not meaningfully change the results.

2. I struggled a little bit to wrap my head around the definition of accuracy in the test condition (Figs 3,5,8), wondering whether it was defined as performance on the first trial in each test context (which is the most indicative) or over the entire course of the test condition (where subjects should know the correct goal within a context after at most 1-3 trials depending on the experiment, since goals were deterministically rewarded). Page 34 suggests that it's only these first trials, which differentiate model predictions, so I would just ask for clarification about the measure used.

The measure of accuracy in Figures 3, 5, and 8 is accuracy over all of the trials. This metric is not meant to differentiate the predictions, but as a check that both the models and human learn the task and do not select goals randomly. We have clarified this in the figure label:

Figure 3:

Goal accuracy of models (light grey) and human subjects (dark grey) across all trials in the training and test contexts. Chance performance denoted with dotted line.

Figure 5:

Goal accuracy of models (light grey) and human subjects (dark grey) across all trials in the training and test contexts. Chance performance denoted with dotted line.

Figure 8:

Goal accuracy of models (light grey) and human subjects (dark grey) across all trials in the training and test contexts. Chance performance denoted with dotted line.

3. Relatedly, I would ask to place a description of the test condition in a more prominent location with information about how many trials (as in table S1-3) and perhaps the sampling across test conditions. At present, this information is only available from a few different locations in the manuscript. Since this is all quite similar across experiments a brief description of test design and measures used for participants could be placed somewhere between the Results heading and the Experiment 1 heading.

We have added tables 1-3 to the results section to address this point.

4. This paper, which explores blends of multi-task policies, might be of interest: Saxe et al. (2017) Hierarchy through composition with multi-task LMDPs; ICML

We agree that this is an interesting comparison. We have added the following on page 23-24:

A particularly interesting proposal is that a composition of multiple, simultaneous actions using a linear approximation to the Bellman equation can result in rapid generalization to a change in rewards (Todorov, 2007; Saxe, et al., 2017). These linear approximations have recently been argued to be biologically plausible implantation of planning, consistent with behavioral data and hippocampal recordings (Piray & Daw, 2019).

Very minor

1. Fig 9 labels for A and B are different (1>4 vs T1 vs T4), even though they represent the same contrast.

2. **Page 10, second paragraph. It's unclear to me what is meant by "task domain".**

We have addressed the above points in the manuscript.

Finally, I would like to commend the authors for making all code and data public.

Reviewer #2: The authors aim at investigating the computational processes that lead to generalization in human reinforcement learning. In particular, they contrast different models that implement joint, dis-joint and meta-generalization. There are many things to like about this paper. The tasks are very clever, the sample sizes are above average, the very elegant modeling and the important research question. I have reviewed this at another journal and I noted that in the present version of the manuscript, the authors addressed all the concerns I expressed in the first round of reviews (including additional simulations, better free parameters estimation and model-comparison) and also amended the discussion. I am happy to recommend for publication as is.

We thank the reviewer for their encouraging comments and all of their helpful feedback in previous reviews.

Reviewer #3: This paper investigates whether humans generalize task components in a conjunctive or independent manner as a function of the statistical structure of the task environment. The study describes three experiments in which participants were asked to navigate a grid world to a rewarded goal state. On each trial, participants had to identify one out of two valid mappings from keys to directions (mappings) to reach the relevant goal state (goals). The generalizable components of a task are operationalized as the correct mapping and the correct goal, respectively, and are both contingent on an observable context. Task structure is operationalized as the mutual information between mappings and goals, and varied across experiments. The study derives qualitative behavioral predictions for each experiment from three different non-generalizing agents, as well as three different generalizing agents described in earlier work (Franklin & Frank, 2018). The "joint" generalizing agent clusters contexts based on the joint statistics between goals and mappings. The "independent" generalizing agent assigns contexts to clusters that represent goals and mappings separately. The meta-generalization ("mixture") agent arbitrates between joint and independent clustering. Computational results indicate that the optimal type of clustering (joint, independent, mixture) depends on the task structure, that is, the joint agent performs well in environments with high mutual information between goals and mappings whereas the independent agent performs well in environments with low mutual information between goals and mappings. The task structures deployed across the three experiments were found to arbitrate between the generalizing models. The manuscript concludes that behavioral results are overall consistent with a mixture of joint and independent clustering and that participants adapt their clustering strategy as a function of mutual information between task components.

The present manuscript nicely complements preceding work by contrasting human generalization performance against performance of three generalization models introduced by Franklin & Frank (2018). I believe that the scientific questions addressed by this study lie well within the scope of this journal and that the manuscript could be an interesting addition to the literature on human generalization and reinforcement learning. Moreover, I believe that the computational analyses and experimental setup are well suited to investigate how humans generalize task components as a function of statistical structure. I particularly commend the authors for their rigorous treatment of outlier detection and subject exclusion in their MTurk study. Overall, I found the manuscript to be well-written and enjoyed reading it.

The manuscript touched on most of my concerns in the Limitations section of the Discussion. However, remaining major concerns regard the generality of the study's results with respect to blocked vs. interleaved training (Major Comment 1), a dissociation between mixed clustering within individuals vs. mixed clustering between individuals (Major Concern 2), the conclusiveness of difference scores (Major Comment 3) and the discussion of related work in machine learning (Major Comment 4). That said, I believe that all comments are addressable in a revision.

Below I provide a list of more detailed comments that will hopefully be useful in improving this manuscript.

We thank the reviewer for their thoughtful feedback and encouraging remarks.

Major Comments

1) Generality of joint clustering (blocked vs. interleaved training): Results from experiment 1 and 3 are taken as evidence that participants cluster task components jointly when there is high mutual information between task components. However, I am wondering whether this finding is contingent on the design choice that "contexts were autocorrelated in time" (p. 35). Recent experimental work indicates that such blocked training promotes the learning of factorized representations (e.g. for clusters of task components) whereas interleaved training impairs such acquisition (Flesch, 2018). However, if I understand correctly, then the joint agent, as well as the meta-generalization agent appear to be indifferent to this manipulation, that is, their clustering behavior should be independent of whether contexts are presented in a blocked or interleaved fashion. The reader may suspect that participants would show little to no evidence for joint clustering when task contexts are interleaved instead of blocked. This may be due to increases in memory demand, as touched on by the authors in the discussion. Thus, to conclude that participants show a general tendency for joint clustering when there is mutual information between task components, the manuscript would benefit from an additional version of experiment 1 with interleaved contexts in the training phase. Alternatively, the potential effects of blocked vs. interleaved training on structure learning could be treated in the Limitations section of the manuscript.

We agree with the reviewer that the Flesch paper is relevant and that context order is likely very important in learning these tasks. It is not clear to us whether joint and independent clustering would be differentially affected in human subjects. There is no qualitative difference in our computational modeling, as the approximate inference method we use (an approximate global MAP) is insensitive to these manipulations (as opposed to, for example, a local MAP). However, we note that neural network model implementations of context clustering have assumed a gating process in which prefrontal cortical neural populations are updating when the abstract structure changes (Collins & Frank 2013). Notably, like the empirical data from Flesch, the development and generalization of abstract structures in these gating networks is actually enhanced during block training (Rougier et al, 2005), amounting to non-exchangeable approximate inference (Schulz et al., 2018). However we have not explored this manipulation in the context of joint vs independent clustering.More generally, we think running additional experiments is beyond the scope of the current work, as an investigation of training curriculum in generalization merits its own study (indeed, one of the authors has ongoing research to this effect).

We have added the following to the limitations section on page 25:

> *A related limitation is the auto-correlated presentation of training contexts in the tasks. While the approximate inference method used in the computational modeling (an approximate global MAP estimate) is generally insensitive to order manipulations, this does not appear to be true for humans subjects, who show substantial order effects in both learning (Flesch et al., 2018) and in generalization tasks (Schulz et al., 2018). Moreover, we note that neural network model implementations of this context clustering have assumed a gating process in which prefrontal cortical neural populations are updating when the abstract structure changes (Collins & Frank 2013). Notably, the development and generalization of abstract structures in these gating networks is actually enhanced during block training (Rougier et al, 2005), as in the empirical data; this amounts to non-exchangeable approximate inference (Schulz et al., 2018). However we have not explored this manipulation in the context of joint vs independent clustering. We would expect this pattern to extend here as well, but how the training regime interacts with meta-generalization is unclear.*

2) Meta-generalization vs. mixture of joint and independent agents in experiment 3: Results from experiment 3 are taken as evidence that participants' behavior conforms best with the meta-generalization agent. However, the qualitative effects associated with the independent clustering strategy (contrast between test contexts 1 and 4), on the one hand, and the qualitative effects indicative of joint clustering (contrast between test contexts 2 and 3), on the other hand, may be driven by different subpools of participants. That is, instead of each participant adapting a mixture of independent and joint clustering (as suggested by the meta-generalization agent), the observed effects may be explained by a mixture of one set of participants adopting an independent clustering strategy (thus contributing to the observed contrast between test contexts 1 and 4) and a different set of participants adopting a joint clustering strategy (contributing to the observed contrast between test contexts 2 and 3). Behavioral analyses at the group level permit claims about individual subjects, as acknowledged by the authors (p. 34). However, dissociating these two hypotheses may not require fitting a model to each individual subject. Instead, one could

statistically investigate whether evidence for the joint strategy (performance difference between test contexts 2 and 3) and the independent strategy (performance difference between test contexts 1 and 4) is driven by different subpools of participants.

We agree with the reviewer that this is a relevant question but our modeling suggests that we are limited in our ability to detect a difference between a mixture of joint and independent generalization vs. individual subjects relying on both strategies within a single task. We would also note that even if individuals are using a single strategy within a task, they may still use a meta-learning strategy across tasks. As such, two pools of subjects using different strategies could reflect individual subjects converging on a single strategy through a meta-learning process.

Nevertheless, we would be able to detect a mixture of joint and independent subjects through a negative correlation in their difference scores (test context 1 vs. 4 and test contexts 2 vs. 3). We found no correlation between these two scores, and while we don't want to over-interpret a null, we at minimum fail to find evidence for different pools of subjects driving each effect. We examined other metrics, such as the co-occurrence of each effect at all, and could not differentiate between the two models.

We have added the following to our discussion on page 26:

As a result, it is difficult to make claims of whether individual subjects show a mixture of joint and independent clustering in experiment 3, or whether our findings are driven by two separate pools of subjects. We note that this distinction does not speak to whether individual subjects use a meta-generalization strategy within a given task, as a meta-generalization that deterministically chooses a strategy would produce two pools of subjects. At a group level, we would expect to see a negative relationship between the joint and independent effect if two separate pools of subjects drive the effects, a relationship we fail to see (see SI:Meta-Generalization vs. Mixture of subjects*).*

We have added the following to the supporting information "Meta-Generalization vs. Mixture of subjects":

The results presented for experiment 3 could have plausibly been either individual subjects adopting a meta-generalization strategy or a mixture of subjects performing only one strategy. In order to assess this, we examined the difference scores for individual subjects to see if there was a relationship between a subjects tendency to show the independent effect (greater accuracy in test context 1 than 4) and their tendency to show the joint effect (greater accuracy in test context 2 than 3).

We also examined the relationship between the two difference scores in the model, comparing the meta-learning agent to an equal mixture of joint and independent clustering agents. As in all of our simulations above, we drew 200 batches of simulated tasks samples from a pool of 2500 and match the sample size to that of human subjects.

We first looked for a correlation between the two difference scores. Intuitively, we would expect a negative relationship between the difference scores in a mixture of agents, as an agent would either show one effect or the other. This bears out in our modeling where there was a negative correlation in the mixtures of joint and independent agents (mean r=-0.17), with 98.5% of batches showing this negative correlation. Surprisingly, there was also a modest negative correlation in our batch of the meta-agent (mean r=-0.07), but this effect was not statistically reliable as 77.5% of batches had r-values below zero. Our human subject data, in contrast, showed a mild positive correlation that was not statistically distinguishable from zero (r=0.04, p>0.3), thus presenting a null result for the mixture of joint and independent agents.

3) Conclusiveness of difference scores: Admittedly, I found it difficult to draw conclusions about each agent's behavior from the difference scores (e.g. Fig. 4). It might help to break down each agent's performance as a function of experiment condition (e.g. accuracy of the joint vs. independent agent as a function of switch condition in experiment 1). For instance, to illustrate the benefit of the independent vs. the joint model in experiment 3, the reader might want to compare absolute performances between two models in test context 1 (I assume that the independent model is predicted to perform better than the joint model), as well as in test context 2 (here, I assume the joint model is predicted to perform better than the independent model), rather than just comparing performance between conditions within each agent and comparing overall performance between agents. Moreover, to provide further evidence of joint clustering in human participants, one would expect that participants would approach the goal predicted by the joint clustering model on the very first trial of a test context in the switch condition of experiment 1 (as soon as participants figure out the correct mapping). That is, on the first trial of the test context with a red mapping in the switch condition of experiment 1 (c.f. Fig. 3), the joint clustering model would predict that participants mistakenly approach goal A more than goal B, as the former was clustered with the red mapping in the training condition. If I am not mistaken, then this would be a testable prediction of the joint clustering model that seems worth an assessment over and above a performance difference between repeat and switch conditions across all trials.

In previous presentations of these data, we have found that presenting each agent's performance as a function of the contexts to be difficult to process for readers and as such, we are hesitant to add this. There are many contexts, and it can be difficult for the reader to follow; moreover there are many individual differences factors that drive changes in overall performance. The key diagnostic predictions that differentiate the models lie in the difference scores, and we believe this to be the most appropriate measure to report.

We do agree with the reviewer that the behavior in the first trial of the test contexts can be diagnostic. We did not find compelling evidence that in the switch condition, subjects were more likely to choose goal A over goal B with the red mapping (mean = 43.9%, $p<0.91$), even when we restrict the analysis to the very first test trial to rule out learning effect during the test phase (mean = 59.3%, $p<0.23$). This analysis is likely underpowered, and we have opted not to include it in the manuscript.

More generally, in the first test trial, we would expect to see evidence of positive/negative transfer (in terms of absolute accuracy) for effects of test condition if subjects are adopting a joint

strategy. We find evidence for positive transfer in the repeat condition (mean accuracy = 72.5%, $p<0.001$) but do not find evidence of negative transfer in the switch condition (mean accuracy = 46.9, $p<0.78$).

4) Related work in machine learning: There are related approaches in machine learning targeting generalization across task environments, such as hierarchical reinforcement learning and clustering of action sequences (e.g. Thrun & Schwartz, 1995), zero-shot task generalization (e.g. Oh et al., 2017) or generalization through state space clustering (e.g. Hashemzadeh, Hosseini & Ahmadabadi, 2019; Mannor et al., 2004). Discussing the relationship to some of these ideas, and delineating the proposed agents from existing approaches would make this paper more accessible to the machine learning community.

We have addressed this comment with the following additional text in the Introduction on page 3:

> *We note that this desideratum is not limited to clustering models and there are many possible decompositions of the reinforcement learning problem for the purpose of generalization more broadly (Da Silva et al., 2012; Dietterich, 2000; Sutton et al., 1999; Thrun & Schwartz, 1995). Often, task components have been derived from a decomposition of the value function over actions and states. Here, we consider a decomposition of the learning problem into the two types of information that determine the value function, information about movement through an environment (the ``transition function'') and learning information about rewards or goals (the ``reward function'').*

And the following in the Discussion on page 21-22:

> *Previous empirical work has provided support for clustering models as an account for generalization of both operant (Collins et al., 2014; Collins & Frank, 2016) and Pavlovian values (Gershman & Hartley, 2015; Gershman et al., 2013). This approach to generalization is qualitatively similar to those found in the machine learning literature that rely on policy re-use in multi-task settings (Mahmud et al., 2013; Rosman et al., 2016; Wilson et al., 2012). Alternatively, an agent may make inferences about unseen portions of the task space solely relying on information present in the task, either through clustering portions of the task space (Hashmezadeh et al., 2019; Mannor et al., 2004) or by function approximation (Konidaris et al., 2011). Humans appear to both cluster the task state for the purpose of planning (Tomov et al., in press)and interpolate rewards continuously in unvisited states (Wu et al., 2018), and moreover, generalize within and across tasks simultaneously (Schultz et al., in press).*

And the following in the Limitations on page 23:

> *Moreover, planning is computationally expensive and poorly understood. It has been shown in purely computational agents that generalize parameterized skills (sequential policies) can reduce exploration costs in novel domains (Oh, et al., 2017), and we might expect human subjects to generalize similarly.*

Minor Comments

1) General: The manuscript would benefit from a proper definition of goal popularity and mapping popularity as experiment factors. Similarly, it might be worth adding a proper definition of the switch vs. repeat conditions to the statistical analysis section.

We have added definitions of goal popularity in tables 1-3. This statistical analysis section already contains a formal description of the switch vs repeat conditions, from page 48:

> "*[Experiment 1] was modeled with the following logistic regression model:*
>
> $$logit(p) = \beta_s S + \beta_H H + \beta_t t + \beta_{rep}\, rep$$
>
> *where* $S \in \{0, 1\}$ *is equal to 1 for the (between-subjects) ``Switch'' condition, where ``H''* $\in \{0, 1\}$ *is equal to 1 when the rewarded goal for the context is the high popularity goal."*

2) General: To facilitate interpretation of agent behavior for the reader, it would be helpful to depict each agent's expected clustering in the form of Fig. 2 for each experiment.

Panels B in figures 5 and 8 show the expected goal clustering under the joint and independent clustering models. We have added a similar panel B to figure 3 to aid with this.

3) P. 4-5: The intuition behind the tradeoff is still at bit unclear at this point. It might help to include an example.

We have added the following analogy to clarify the tradeoff on page 3:

> *As an analogy, because clustering rewards and transitions independently ignores the relationship between them, it brings a similar set of benefits in limitations as a Naive Bayes classifier. Information will be lost, but this may result in a more robust statistical model (Friedman 1997). In contrast, joint clustering will, with sufficient experience, learn the correct generalization statistics and a potential cost of sample efficiency.*

4) Figure 2: The depiction of goal values and transitions on the right side appears to be redundant with Figure 1.

We agree that this is redundant but we believe that this improves the readability of the figure.

5) P. 12, "Each model was simulated with sampled parameters on 2500 random instantiations of the tasks": I'm wondering why the models weren't simulated on the exact same instantiations of the tasks that the human subjects performed to ensure proper comparison with human data?

Our simulations allow us to estimate how the probability that a randomly sampled experiment would have shown the effects under each of the computational models (i.e. what one would expect to see in the long run). The simulations the reviewer is suggesting would allow us to estimate the probability that a randomly sampled set of model instances will show the effects of interest. In expectation, these two are the same, and while there may be differences in the magnitude of the effects, we believe either to be appropriate.

We have added the following clarifying statement after the cited passage on page 28:

> *This allows us to estimate the probability that a randomly sampled experiment would produce the observed effects.*

6) P. 33: Model-based planning appears to be associated with a cognitive cost that participants arbitrate against the benefits of planning (Kool, Gershman, Cushman, 2017). Following this intuition, the discussion mentions memory demand as an alternative explanation to the meta-generalization agent. It might be worth to highlight in this context that a higher number of trials in the last two experiments (relative to experiment 1) could have led participants to adapt independent clustering as opposed to joint clustering (thus, explaining lesser evidence for joint clustering in experiments 2 and 3 compared to experiment 1).

We have added this point to our limitations following our discussion of related work on the cost of control (page 24):

> *Because subjects tend to extend less cognitive effort in more demanding tasks (Kool et al., 2010, 2017; Westbrook et al., 2013), it is possible that they relied on a less cognitively demanding strategy. Relatedly, the higher number of trials in the latter two experiments relative to the first may have encouraged subjects to adopt a more cognitively demanding strategy, as they may have changed the relative costs and benefits of doing so.*

7) P. 42: If I understand correctly, then the two nuisance factors (number of trials, context repetition) are supposed to capture learning within the test phase. However, human learning may not just be a function of context repetition, but whether participants reached the correct goal state in the previous trial. Therefore, the authors may want to consider including a nuisance factor coding for the interaction between context repetition and accuracy on the previous trial.

We have changed the nuisance regressors in our statistical analysis from including an indicator for whether a context is repeated to whether the interaction of whether a context is repeated and the previous goal choice is correct. This adds "stickiness" to the participants' choices and does not meaningfully change the results.

Typos

1) P. 4. “movement through and environment” --> “movement through an environment”

2) P. 6: “such that is was not possible to learn” --> “such that it was not possible to learn”.

3) P. 13: The text seems to incorrectly reference Fig. S3A (instead of Fig. S2A)

4) P. 29: “expect these tasks to produces” --> “expect these tasks to produce”

5) P. 49: “and allowed for the and allowed for” --> “and allowed for”

6) Experiment 3 appears to be missing a subsection heading “Computational Modeling”

> *These have been addressed. We were missing a supplemental figure (S2 in the revised manuscript) and have included it to address point 3 above.*

References

Flesch, T., Balaguer, J., Dekker, R., Nili, H., & Summerfield, C. (2018). Comparing continual task learning in minds and machines. Proceedings of the National Academy of Sciences, 115(44), E10313-E10322.

Franklin, N. T., & Frank, M. J. (2018). Compositional clustering in task structure learning. PLoS computational biology, 14(4), e1006116.

Hashemzadeh, M., Hosseini, R., & Ahmadabadi, M. N. (2019). Clustering subspace generalization to obtain faster reinforcement learning. Evolving Systems, 1-15.

Kool, W., Gershman, S. J., & Cushman, F. A. (2017). Cost-benefit arbitration between multiple reinforcement-learning systems. Psychological science, 28(9), 1321-1333.

Mannor, S., Menache, I., Hoze, A., & Klein, U. (2004). Dynamic abstraction in reinforcement learning via clustering. In Proceedings of the twenty-first international conference on Machine learning (p. 71). ACM.

Oh, J., Singh, S., Lee, H., & Kohli, P. (2017). Zero-shot task generalization with multi-task deep reinforcement learning. In Proceedings of the 34th International Conference on Machine Learning-Volume 70 (pp. 2661-2670). JMLR.
