# Supplementary material for: Generalizing to generalize: Humans flexibly switch between compositional and conjunctive structures during reinforcement learning
Source: PLoS Comput Biol. 2020 Apr 13;16(4):e1007720. doi: 10.1371/journal.pcbi.1007720 (PMC7179934; doi:10.1371/journal.pcbi.1007720)
Supplement: Attachment — Submitted filename: Franklin & Frank_2019.docx [file pcbi.1007720.s014.docx]

This paper investigates whether humans adaptively change their generalization strategies depending on environmental statistics. More specifically, the hypothesis is that participants will change the degree to which they blend “joint” and “independent” clustering of the task-relevant statistics (action set and reward function). The paper describes a series of three experiments which vary in the degree to which participants should favour the joint (exp 1), independent (exp 2) or meta-generalization (exp 3) strategies. The results show that humans are biased to generalize even when this is not advantageous, with the models further indicating a meta-generalization strategy.

The study is designed to address an important and timely question: What are the mechanisms which enable humans to flexibly generalize behaviour to novel situations? The tasks are well-designed, and the authors take great care to rule out lower-level confounds. The simulations are done very carefully with state of the art techniques. Overall, I think this is a well-crafted manuscript with nothing major standing in the way of publication.

That being said, however, I think the paper in its current form under-utilizes the rich behavioural data from the training and test conditions. I have a few suggestions which I think might ultimately aide understanding of the paper’s conclusions and methods and provide richer information about human behaviour than from the mostly qualitative model comparison.

1. A key component of the model in the paper is the clustering which is done via a Chinese Restaurant Process. This made me wonder how essential this component is to explain the data. It makes the key prediction that accuracy and learning speed are influenced by context popularity, rather than frequency of trials. However, I don’t see the alternative “frequency-based” models tested – the flat models do not work across contexts, so ask a different question. I wondered to what extent a Q-learning agent which averages over all trials would predict participant’s behaviour. For instance, the Q-learner might average across all of participants’ trajectories (rather than contexts) and thus make different predictions to a clustering model based on “popularity”. Even though each goal was rewarded the same amount of trials, participants’ choices likely did not reflect this (~75-80% performance). In this sense, a goal that was more frequently visited (even when unrewarded) might be chosen with higher likelihood in the test conditions. I don’t expect this model to do very well, but I nevertheless think it would be an appropriate control model that would add additional confidence in the results.
2. I wondered in how far the first few button presses (rather than a trial overall) could be indicative of generalization strategy. First, did participants show a preference for either one of the hands on the first button press? I ask this, because some mappings were more popular on a context level (though not on overall trials), so we might expect participants to be biased in some way. More generally, participants might have acted consistent with a) navigating to the most popular goal and b) the mapping for which this goal was more popular, e.g. in experiment 2, we might expect them to use the left hand, because goal A was more frequent overall and more frequently associated with the left hand. Thus, a joint-clustering agent should be biased to “try” the mapping with higher joint likelihood. After this initial button press, participants can reason conditional on the correct mapping. Thus, if the chosen mapping was correct, participants should continue navigating towards the same goal. However, if the mapping was incorrect, it would then be the second button press which would distinguish joint from independent clustering. If participants then press the button (on the other hand) which corresponds to the same direction as their first button press, then they likely clustered independently (still navigating to the same goal), but if they choose a different direction they likely clustered jointly (on cases where the two mappings have different goal popularities, e.g. exp 1 and 3). Perhaps there is not a lot of data to work with for this, but I think it would give a strong indication of the strategies employed by participants.
3. A similar point relates to how well each strategy predicts behaviour in the training condition. One of the paper’s central claims is that humans adaptively adjust their generalization strategies according to the task statistics. In this sense, the models should make predictions about behaviour in, say, context 3, after having seen contexts 1 and 2 for some limited amount of time (due to the autocorrelation). If the strategies are adaptive, one might expect participants behaviour to be gradually better predicted (even within training) by one strategy than the others.
4. Did the models also take into account progression through the test conditions? This would change goal popularity and would lead to different predictions about behaviour – which might partly explain the different pattern of participants’ goal choice in experiment 2 (Fig 7c and d).

Minor:

1. How likely were participants to simply repeat their previous choice of goal in a new context? Does including this regressor at all affect the model parameters in the logistic regression and the Bernoulli analysis? It seems quite likely that in new contexts there would be at least some “stickiness” to participants’ choices which gives way via reinforcement to the correct goal over time, but perhaps dominates somewhat on the first trial in any new test condition.
2. I struggled a little bit to wrap my head around the definition of accuracy in the test condition (Figs 3,5,8), wondering whether it was defined as performance on the first trial in each test context (which is the most indicative) or over the entire course of the test condition (where subjects should know the correct goal within a context after at most 1-3 trials depending on the experiment, since goals were deterministically rewarded). Page 34 suggests that it’s only these first trials, which differentiate model predictions, so I would just ask for clarification about the measure used.
3. Relatedly, I would ask to place a description of the test condition in a more prominent location with information about how many trials (as in table S1-3) and perhaps the sampling across test conditions. At present, this information is only available from a few different locations in the manuscript. Since this is all quite similar across experiments a brief description of test design and measures used for participants could be placed somewhere between the Results heading and the Experiment 1 heading.
4. This paper, which explores blends of multi-task policies, might be of interest: Saxe et al. (2017) Hierarchy through composition with multi-task LMDPs; ICML

Very minor

1. Fig 9 labels for A and B are different (1>4 vs T1 vs T4), even though they represent the same contrast.
2. Page 10, second paragraph. It’s unclear to me what is meant by “task domain”.

Finally, I would like to commend the authors for making all code and data public.
